# Modeling the Risk of Secondary Malignancies after Radiotherapy

**DOI:** 10.3390/genes2041033

**Published:** 2011-11-29

**Authors:** Uwe Schneider

**Affiliations:** 1 Vetsuisse Faculty, University of Zurich, Zurich 8057, Switzerland; E-Mail: uwe.schneider@uzh.ch; Tel.: +41-62-8367805; Fax: +41-62-8367801; 2 Radiotherapy Hirslanden AG, Rain 34, Aarau 5000, Switzerland

**Keywords:** radiation induced cancer, radiotherapy, second cancer

## Abstract

In developed countries, more than half of all cancer patients receive radiotherapy at some stage in the management of their disease. However, a radiation-induced secondary malignancy can be the price of success if the primary cancer is cured or at least controlled. Therefore, there is increasing concern regarding radiation-related second cancer risks in long-term radiotherapy survivors and a corresponding need to be able to predict cancer risks at high radiation doses. Of particular interest are second cancer risk estimates for new radiation treatment modalities such as intensity modulated radiotherapy, intensity modulated arc-therapy, proton and heavy ion radiotherapy. The long term risks from such modern radiotherapy treatment techniques have not yet been determined and are unlikely to become apparent for many years, due to the long latency time for solid tumor induction. Most information on the dose-response of radiation-induced cancer is derived from data on the A-bomb survivors who were exposed to γ-rays and neutrons. Since, for radiation protection purposes, the dose span of main interest is between zero and one Gy, the analysis of the A-bomb survivors is usually focused on this range. With increasing cure rates, estimates of cancer risk for doses larger than one Gy are becoming more important for radiotherapy patients. Therefore in this review, emphasis was placed on doses relevant for radiotherapy with respect to radiation induced solid cancer. Simple radiation protection models should be used only with extreme care for risk estimates in radiotherapy, since they are developed exclusively for low dose. When applied to scatter radiation, such models can predict only a fraction of observed second malignancies. Better semi-empirical models include the effect of dose fractionation and represent the dose-response relationships more accurately. The involved uncertainties are still huge for most of the organs and tissues. A major reason for this is that the underlying processes of the induction of carcinoma and sarcoma are not well known. Most uncertainties are related to the time patterns of cancer induction, the population specific dependencies and to the organ specific cancer induction rates. For radiotherapy treatment plan optimization these factors are irrelevant, as a treatment plan comparison is performed for a patient of specific age, sex, *etc.* If a treatment plan is compared relative to another one only the shape of the dose-response curve (the so called risk-equivalent dose) is of importance and errors can be minimized.

## 1. Introduction

In developed countries, more than half of all cancer patients receive radiotherapy at some stage in the management of their disease. Advances in cancer treatment have steadily improved survival times over the past three decades. In 1971, three million people were living with cancer, representing approximately 1.5% of the US population. In 2001, already ten million people were cancer survivors, approximately 3.5% of the US population [1]. In the absence of other competing causes of death, 64% of adults whose cancer was diagnosed during 1995 and 2000 could expect to be alive 5 years after diagnosis, compared with 50% for those whose cancer was diagnosed during 1974 and 1976. Among children, the improvement of cure rates is even more pronounced with 79% five-year cancer survivors (1991-2000), compared with 56% after diagnosis during 1974 and 1976 [1].

Among all cancer survivors in 2001, 14% had received a cancer diagnosis more than 20 years ago [1]. Approximately half of these long-term survivors received a radiotherapy treatment and are thus subject to radiation-related side effects. These long-term survivors experience a significant incidence of chronic health problems after their treatment, including second primary cancer [2]. A second cancer is defined as a histologically distinct cancer that develops after the first cancer. In total, 95,000 of the 1.2 million new cancers diagnosed every year in the United States are second cancers. Second cancers therefore account for 6%–10% of all cancer diagnoses and are the fourth or fifth most common cancer in the United States [3]. A radiation induced secondary malignancy can be the price of success, if the primary cancer is cured or at least controlled.

Research and development in radiation oncology is mainly directed to further increase cure rates. This is currently achieved by the application of new radiation treatment modalities such as intensity modulated radiotherapy (IMRT), intensity modulated arc-therapy, proton and heavy ion radiotherapy. With the application of these treatment techniques also a larger number of secondary cancers are expected for two reasons. One consequence of improved cure rates is a further increase in the number of long-term survivors who are at risk of developing a second cancer. It is believed by some researchers that we will see an increase in second malignancies due to the substantial increase in beam-on time of IMRT techniques to deliver the same target dose but a different distribution of dose (“low dose to a large volume”) compared to conventional treatment techniques [4,5]. In addition, during proton and heavy ion radiotherapy, neutrons are created and could also have an impact on second cancer induction. Therefore, it could be of great importance to know the risk for the patient to develop a cancer, which could be caused by the application of a potential new radiation treatment.

The long term risks from modern radiotherapy treatment techniques have not yet been determined and are unlikely to become apparent for many years, due to the long latency time for solid tumor induction. Therefore, decisions will have to be made using alternative types of evidence, as is common in many other rapidly advancing disciplines of medicine. The evidence will, by necessity, comprise of theoretical predictions [6]. Therefore there is a need to develop models for risk assessment based on the current knowledge of radiation induced carcinogenesis.

## 2. Risk Factors for Second Cancers

There are several known risk factors for second cancers. Host-related risk factors for the induction of second malignancies are age at diagnosis, gender and treatment of primary cancer. Younger age at diagnosis of the primary cancer is associated with an increased risk of a second cancer, primarily among radiation-associated second cancers [3,7-9]. The reasons for these age effects might be related to one or more of the following: increased susceptibility of the underlying tissue to the mutagenic effect of therapy at a younger age; the higher rate of cell proliferation during the early stages of development; genetic susceptibility or a longer period of follow-up of the childhood cancer survivor cohort, which allows second cancers with typically long latencies to emerge. The female sex is also associated with an increased risk of second primary cancers, due to the excess number of secondary breast cancers and, to some extent, to the increased occurrence of thyroid cancer in female survivors. Among adults, several studies indicate that, for a given dose of radiation, women are more susceptible to carcinogenesis than men [3].

Certain chemotherapeutic agents, particularly alkylating agents and topoisomerase II inhibitors, increase the risk of developing a second cancer. In some cases, specific genetic changes caused by these agents explain the increased risk of leukemia [3]. Increased risks of solid cancers after Hodgkin's disease have been generally attributed to radiotherapy, since patients receiving combined modality treatment have in general no greater relative risk than patients treated solely with radiotherapy [10]. Only one study has reported a significantly higher risk for solid cancers after combined chemo-and radio-therapy compared with irradiation alone. However, for selected solid cancer sites higher (e.g., lung) or lower (e.g., breast) risks were observed after combined modality treatment than after irradiation alone [10].

Emerging risk factors for second cancers include familial cancer syndromes, gene-environment interactions, lifestyle choices and other medical complications associated with treatment for the primary cancer [3].

Radiation therapy increases the risk of several second cancers in a dose-dependent manner. Most second cancers associated with radiotherapy occur in or near the area that was irradiated and most have a long latency [3,11].

## 3. Epidemiology of Second Primary Cancers after Radiation Therapy

Ionizing radiation can cause most types of cancer, but different organs vary in their susceptibility [2]. The epidemiological data from the Hodgkin's patients treated with radiotherapy and/or chemotherapy indicate that radiation causes mainly solid cancers and chemotherapy mainly leukemia [10]. The risk is highest when the exposure occurs at a younger age [8,9]. In general, cancer risk increases as the total dose of radiation increases and there seems to be a long latency period, probably due to the time required for sufficient mutations to accumulate [3]. Most radiation-associated second cancers develop within or at the edge of the radiation field [11,12].

**Figure 1:**
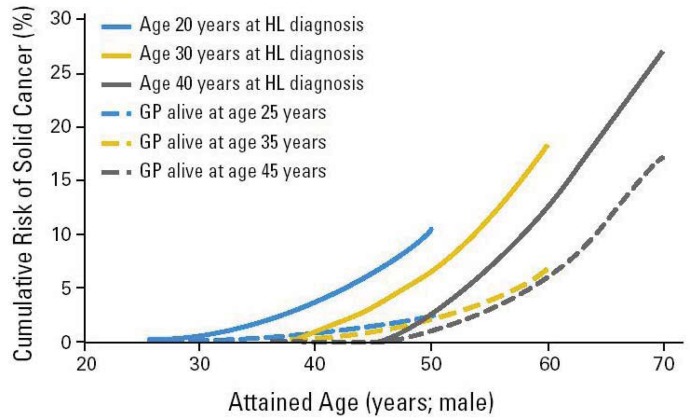
Cumulative risk (solid lines) for a Hodgkin's patient who was treated with radiation- and/or chemo-therapy to develop a second solid cancer as a function of age after diagnosis (attained age). The dashed lines represent the incidence in the general population. Figure is taken from [21].

Radiation-associated bone tumors and sarcomas show all the characteristics of radiation-associated second cancers: there is a clear relationship with radiation dose and the second cancers develop within the radiation field, typically after a latency period of ten years [13]. Another radiation-associated tumor is breast cancer, which has been increasingly reported among patients receiving radiation for Hodgkin's disease [9,14-16]. The latency is typically between 15 and 20 years from primary diagnosis, and the risk is highest among patients diagnosed at a younger age, decreasing to that of the general population for patients receiving radiation for their primary cancer after the age of 30 years. The tumors typically develop within or at the edge of the radiation field. Patients receiving radiation to the neck region are at an increased risk of developing thyroid cancers [17,18]. Patients receiving radiotherapy for prostate cancer are at an increased risk of sarcomas, lung-, bladder- and rectal cancer [19]. Brain tumors have been reported following cranial radiation for histologically distinct brain tumors or for prophylaxis or treatment of central nervous system [20]. A huge body of literature on second cancer induction after radiation therapy is available. The epidemiological data are usually obtained for patients who were treated 20 or 30 years ago and thus the dose was in general not recorded precisely enough to reconstruct possible dose-response relationships. Most meaningful risk data were obtained from the Hodgkin's patients treated with radiation (Figure 1). There are only a few publications where case control studies were performed [14-16,22-26] and which could give more insights into dose-response relationships for second cancer induction.

In summary, the following conclusions can be drawn from epidemiological studies:

(i) Radiotherapy patients are at a higher risk of developing a solid cancer than the general population;
(ii) Nearly all secondary tumors occurred in or at the border of the original treatment fields;
(iii) Children have a much larger risk to develop radiation induced cancer;
(iv) The quantification of secondary tumor risk is not trivial due to the missing information on dose distribution.

## 4. The Characteristics of Dose Distributions from Contemporary Radiation Therapy

For intensity-modulated radiotherapy the photon flux of a treatment field is varied perpendicular to the beam direction. This additional degree of freedom allows to shape the dose distribution more precisely than in conventional radiotherapy (3D-CRT) with treatment fields of constant flux.

The realization of IMRT has two consequences regarding cancer induction. First, for the application of an intensity modulated treatment field, the beam-on time is substantially increased in order to deliver the same target dose. Second, the distribution of dose is different when compared to conventional treatment techniques (“low dose to a large volume”). As a consequence, the scatter dose to the patient is increased, but the integral dose approximately unchanged. The integral dose is the measure of the total energy deposited in the patient outside the target volume. In addition, when IMRT is delivered with photon energies above 10 MeV, neutrons are produced in the treatment head. The neutrons could lead to a considerable contribution to the integral dose, in particular, since neutrons have a large quality factor and thus even a small physical dose can result in considerable biological effects.

**Figure 2:**
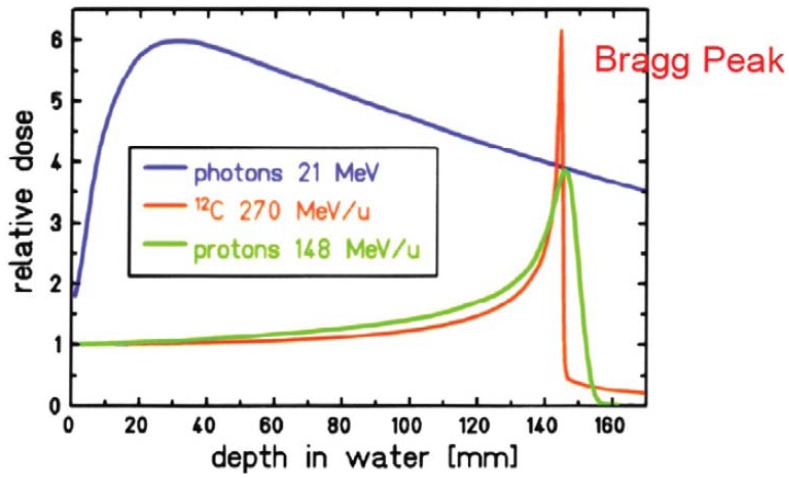
Depth dose distribution for photons and monoenergetic Bragg curves for carbon ions and protons taken from [32].

**Figure 3:**
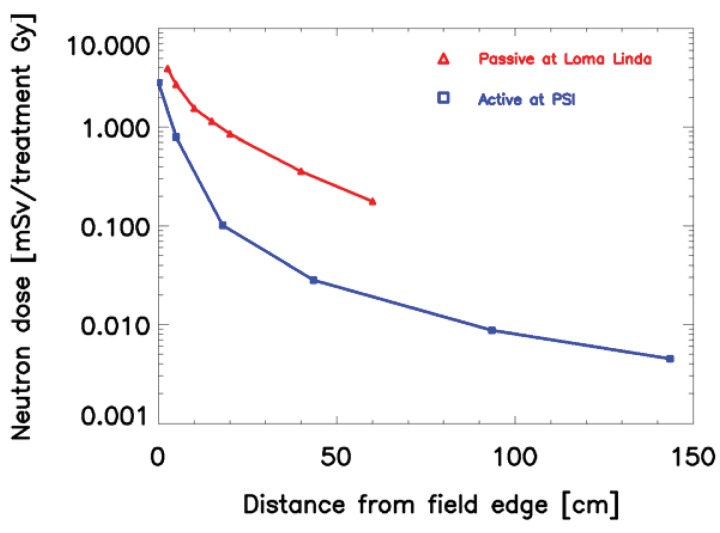
Comparison of neutron dose equivalent given in mSv per treatment Gy for two proton therapy beam lines for a prostate treatment. The red curve shows the results of measurements at the passive (scatter) beam line at Loma Linda [29,30] and the blue curve results from measurements at the active beam line at Paul Scherrer Institute (PSI) [28]. All measurements were performed perpendicular to the beam direction.

When proton or ion therapy is used to treat a patient, the integral dose is lower per se when compared to an equivalent photon treatment. The reason for this is that the dose deposited by protons rises sharply near the end of their range, giving rise to the so-called Bragg peak (Figure 2). This differs significantly from the dose deposition by photons, which is quasi exponential. As a result the integral dose from proton or ion therapy is a factor of 2–3 lower than a comparable photon treatment [27]. This integral dose advantage is somewhat balanced by the additional neutron dose. The nuclear interactions of protons create a halo of neutrons and are responsible for a low dose everywhere in the patient. Two possible beam delivery systems for proton and ion therapy have to be distinguished regarding neutron dose. Active scanning sweeps a fine pencil beam through the target. Since scanning is mainly accomplished by magnetic means, neutrons are mainly produced in the patient itself [28]. In contrast, passively scattered beams are produced by interposing scattering material into a pencil beam to produce a broad beam which covers the whole target volume. Therefore, more neutrons are produced and the neutron dose from scattered beams is much larger than from pencil beam scanning, which is depicted in Figure 3 [29,30]. Close to the target protons offer a distinct advantage due to the lower integral dose. Out-of-field, but within approximately 25 cm from the field edge, the scattered photon dose in intensity modulation turned out to be roughly a factor of 2 lower than the neutron equivalent dose from scattered proton therapy. At larger distances to the field (beyond approximately 25 cm), protons offer an advantage, resulting in doses that are roughly a factor of 2–3 lower [31].

## 5. Modeling Risk of Cancer Induction for Radiotherapy Doses

### 5.1. Simple Models Based on Radiation Protection Concepts

Simple models to predict risk of radiation induced cancer for radiotherapy dose levels are based on conventional concepts from radiation protection, *i.e.*, ICRP [33] or BEIR [34]. These models are based on the linear approximation of the risks of the Atomic-bomb survivors and use effective dose (the tissue-weighted sum of the equivalent doses in all specified tissues) for risk estimation. Basic risk factors are usually modified by a dose and dose-rate effectiveness factor (DDREF) for the application to low dose-rates. The linear model is only valid for doses up to around 1–2 Gy and as such, is in general, not applicable to radiotherapy dose distributions with doses up to around 100 Gy. It is stated by the ICRP, that effective dose is intended for use as a protection quantity on the basis of reference values and therefore is not recommended for epidemiological evaluations, nor should it be used for detailed specific retrospective investigations of individual exposure and risk. Rather, absorbed dose should be used with the most appropriate quality and risk factor data. Organ or tissue doses, not effective doses, are required for assessing the probability of cancer induction in exposed individuals (157 in [33]). It is also the policy of ICRP that it's recommended nominal risk coefficients should be applied to whole populations and not to individuals (81 in [33]).

**Figure 4:**
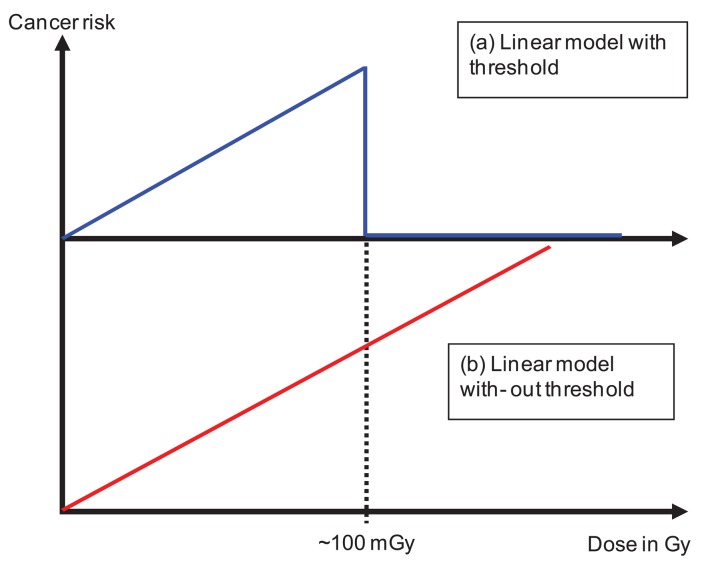
Use of radiation protection cancer risk models in radiation oncology. In (**a**) a linear model is used with a threshold at around 100 mGy in (**b**) the linear model is used over the complete dose range excluding the dose in the target.

Despite all that criticism, radiation protection models were used by several authors. In general they are applied to radiotherapy dose distributions in two different ways:

(i) The dose distribution can be separated into two parts. The primary dose distribution is created by particles impinging on the patient through the opening of the beam aperture. This includes patient scattering mainly produced by Compton scattering (photons) and multiple Coulomb scattering or inelastic nuclear interactions (protons and ions). The scatter dose distribution is generated by radiation scattered from the treatment head, leakage radiation through the collimators and neutrons produced either in the machine or the patient. Radiation protection models can be applied exclusively to the dose originating from scatter radiation [4,35-41]. In principle the linear model is applied to very low doses with a threshold of around 100 mGy. The threshold represents the maximum applied scatter dose during a radiotherapy treatment (Figure 4a). The results of such estimates are that cancer risk is not a function of the integral dose, but proportional to the amount of scatter dose. As a consequence, such studies result in an estimated increase of cancer risk by a factor of 2 to 10 for IMRT and passive proton therapy. The reason for this is the considerably larger amount of scatter and neutron dose of those treatment modalities compared to conventional treatment techniques. While in such situations the application of radiation protection concepts may be appropriate, since exclusively the low doses are investigated, the main disadvantage of such an approach is that the primary dose distribution (>100 mGy) is completely neglected. Thus risk estimates based on scatter dose would only include second cancer induction far away from the treated side. It is reported, however, that only around 20% of all radiation-induced malignancies are found far away from the treated volume [11].
(ii) Other researchers have applied linear models to the complete dose distribution including the primary part (Figure 4b), but excluding the dose delivered to the target volume [42-44]. One result of these studies is that risk estimates are in general proportional to the integral dose and not dependent on the treatment technique (risk after IMRT is approximately the same than after non-IMRT). However, risk was substantially different for different radiation qualities. A considerable risk reduction was observed for protons when compared to photons. While the advantage of these studies is the consideration of the complete dose distribution, the main disadvantage is the application of the linear model to very large doses.

In summary, the application of radiation protection concepts to radiotherapy patients yield contradictory results, since the linear model is applied in two completely different ways to the dose distribution. Risk estimates based on such models have to be applied with extreme care, since above 1 Gy the A-bomb survivor data are better fitted by linear-quadratic or linear-quadratic-exponential models [45,46].

### 5.2. Advanced Models for Cancer Risk Estimates Excluding Fractionation

Despite the large body of experimental and clinical experience which now exists, the general form of the dose-response curve for radiation carcinogenesis following high dose radiation is not completely clear. Experimental and epidemiological studies have yielded a confusing array of dose-response relationships, mainly because the radiation dose is not well known from epidemiological studies.

**Figure 5:**
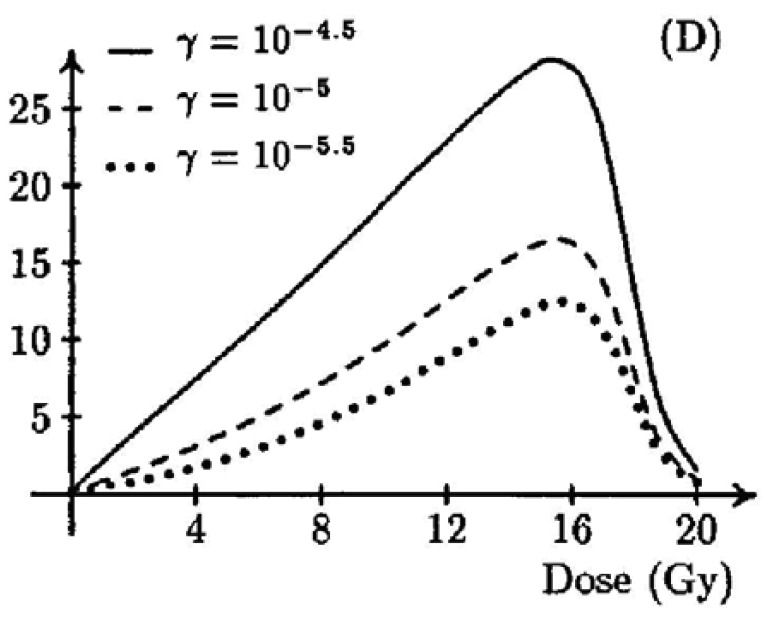
Percentage incidence of carcinogenesis as modeled by Lindsay *et al.* [48] for different values of radiosensitivities γ. Figure is taken from [48].

A two-stage mutation model was used by Wheldon and Lindsay [47,48] to obtain a dose-response curve for doses in the radiotherapy range (shown in Figure 5). They obtained a typical bell shaped dose-response curve with vanishing cancer risk at doses larger than 20 Gy. Their model contained 11 parameters and they studied the parameter dependence in a very general way without obtaining organ specific dose-response functions.

A similar approach was used by Dasu *et al.* [49,50]. They described dose-response relationships with a competition model (cell sterilization *versus* transformations) which takes into account the probability to induce DNA mutations and the probability of cell survival after irradiation. The shape of the curves was analyzed in relation to the parameters. Their results were quite similar to the model of Wheldon and Lindsay [47,48].

Another approach was used by Schneider and Walsh [46] based on the epidemiological data of the A-bomb survivors. They extended the analysis of the A-bomb survivors by including two extra high-dose categories (4–6 Sv and 6–13 Sv) and by an attempted combination with cancer data on patients receiving radiotherapy for Hodgkin's disease. This work provided evidence of a bending over of the solid cancer excess risk dose response curve for the A-bomb survivor at doses larger than 2 Gy. The fit to the A-bomb data provided the model parameters for a bell-shaped dose-response curve for all solid cancers.

It can be concluded that mathematical models of the dose-response curve, not considering fractionation, up to radiotherapy dose levels show a bell shaped behavior due to cell sterilization at large dose. However, epidemiological studies of radiation-induced second cancers in the lung and breast, covering a very wide range of doses, contradict this assumption [50]. A likely resolution of this disagreement comes from considering fractionation effects.

### 5.3. Advanced Models for Cancer Risk Estimates Including Fractionation

Dose fractionation is common in radiation therapy. Fractionation effects can be important for carcinogenesis and can be included into the models by considering cell repopulation between the dose fractions. Repopulation tends to counteract cell killing and accounts for the large discrepancies between the standard model for cancer induction neglecting fractionation at high doses (Section 5.2) and recent second cancer data [51].

Sachs and Brenner [52] and Shuryak *et al.* [53,54] developed dose-response relationships for excess relative risk (ERR) of cancer induction for a wide dose range. Their model integrates, into a single formalism, mechanistic analyses of pre-malignant cell dynamics on two different time scales: short-term during radiotherapy and recovery and long-term during the entire life span. The model was applied to nine solid cancer types (stomach, lung, colon, rectal, pancreatic, bladder, breast, central nervous system and thyroid) using data on radiotherapy induced second malignancies, on Japanese atomic bomb survivors and the statistics on cancer incidence in the general United States population.

**Figure 6:**
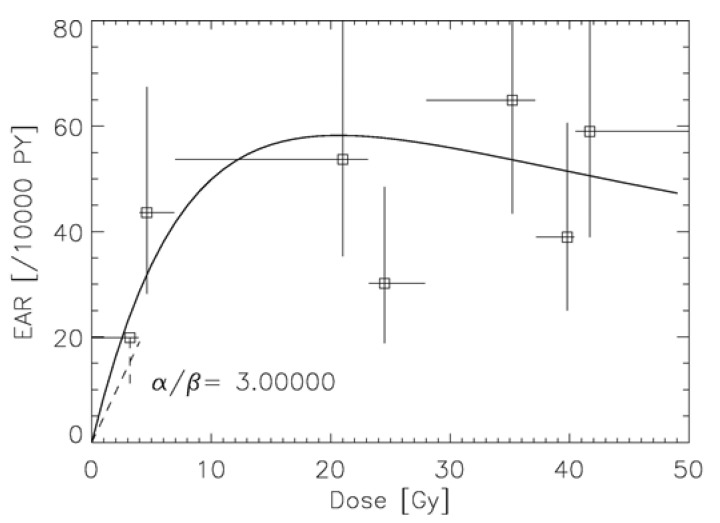
Plot of the modelled excess absolute risk (solid line) to the epidemiological data of Travis *et al.* [11] for α/β = 3 Gy. The dashed line represents the linear model for breast cancer with the corresponding error.

Another approach was developed by Schneider [55] to model excess absolute risk (EAR). The linear-quadratic model of cell kill was applied to normal tissues which are unintentionally irradiated during a cancer treatment with radiotherapy. Tumor induction was modeled such that each transformation process results in a tumor cell. The microscopic transformation parameter was chosen such that in the limit of low dose and acute exposure the parameters of the linear-no-threshold model for tumor induction were approached. The long-term time patterns were modeled exclusively using the A-bomb survivor data. The model describes separately carcinoma and sarcoma induction after fractionated radiotherapy as an analytical function of four parameters and was fitted to 16 solid cancer types: female breast (shown in Figure 6), lung, rectum, colon, mouth and pharynx, stomach, small intestine, liver, cervix, bladder, skin, brain and CNC, salivary gland, bone and soft tissue sarcoma [56-58].

**Figure 7:**
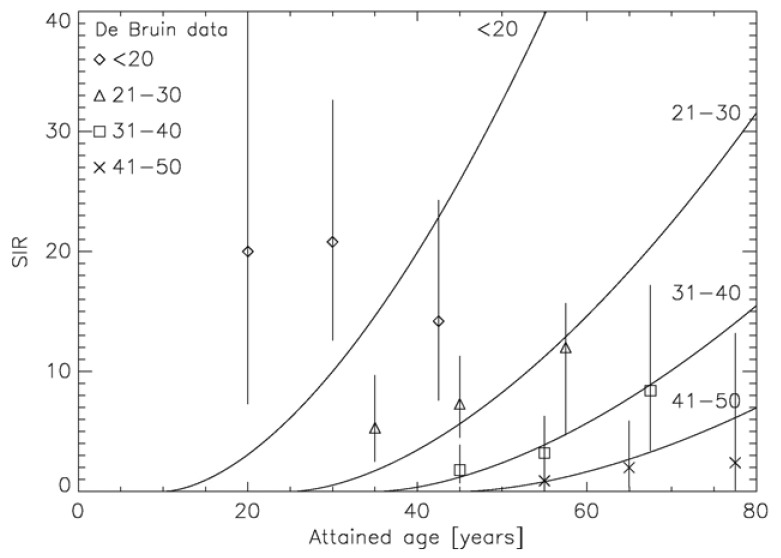
Verification of the modeled time patterns with epidemiological data. Plot of the modeled age dependence of the standardized incidence ratio (SIR) as the solid lines for the age at treatment groups <20, 21–30, 31–40 and 41–50, respectively. The corresponding epidemiological data from de Bruin *et al.* [9] are plotted as the symbols together with the corresponding 95% confidence interval.

The two approaches which include the effect of fractionation into carcinogenesis models use different strategies: Sach and Shuryak model ERR, Schneider EAR. The advantage of modeling ERR is that the transfer of risk estimates between different populations (Japanese-Western) is more robust than using EAR models. However, ERR dose-response relationships require models of the background cancer rate for the different tissues which complicates the model resulting in 11 adjustable parameters. In contrast, EAR models determine directly the probability of a radiation induced cancer. Thus, the presented EAR model needs 4 parameters and uses the time patterns from the A-bomb survivor data, which are shown for breast cancer in Figure 7 [59].

It should be noted here, that the dose response-relationships for radiation induced cancer describe cancer risk as a function of point dose and are in general a non-linear function of dose [45]. As a direct consequence, cancer risk is not proportional to average or integral dose. Unfortunately, in literature cancer risk is often plotted as a function of average dose which is meaningless for doses larger than around one Gy except for linear dose-response relationships.

### 5.4. Concept for Treatment Plan Comparisons with Regard to Cancer Induction

Potentially, the models described in Section 5.4 can be incorporated into radiotherapy treatment planning algorithms, adding second cancer risk as an optimization criterion. Optimization of a radiotherapy treatment usually involves the comparison of different competitive treatment plans for a specific patient. Therefore, it is not necessary to include into treatment planning optimization estimates of ERR or EAR, which are subject to large uncertainties regarding population dependent parameters such as age at exposure, attained age and sex. Treatment planning comparison can be much simplified by using exclusively the dose-dependence of cancer risk models for comparison. Therefore, it makes sense to define a generalized dose parameter which is equivalent to cancer induction and is based on the modeled dose-response relationship for carcinogenesis.

The excess absolute or relative risk in a small volume element of an organ can be factorized into a function of dose RED (risk equivalent dose) and a modifying function *μ* that depends on the variables age at exposure, age attained and sex:

(1) Risk=βRED(D)μ(agex,agea,sex)

where RED is the dose-response relationship for radiation induced cancer and *β* the organ-specific cancer induction rate. The risk equivalent dose RED of a treatment plan is then a three-dimensional distribution of a generalized dose parameter measured in Gy and proportional to risk. RED is organ and tissue depended and thus, for a comparison it should be averaged over each organ and tissue of interest in the patient:

(2) $$\mathrm{OED}=\frac{1}{V_{T}}\sum_{i}V(D_{i})\mathrm{RED}(D_{i})$$

where organ equivalent dose (OED) is introduced which is a dose-response (RED) weighted dose variable averaged over the whole organ volume [60]. It becomes instantly clear that risk ratios for different radiotherapy treatment plans are equivalent to OED ratios which can be simply determined on the basis of the organ specific dose-response relationship (RED) and the dose volume histogram V(D). OED values are keeping the necessary variables and the corresponding uncertainties at a minimum.

**Figure 8:**
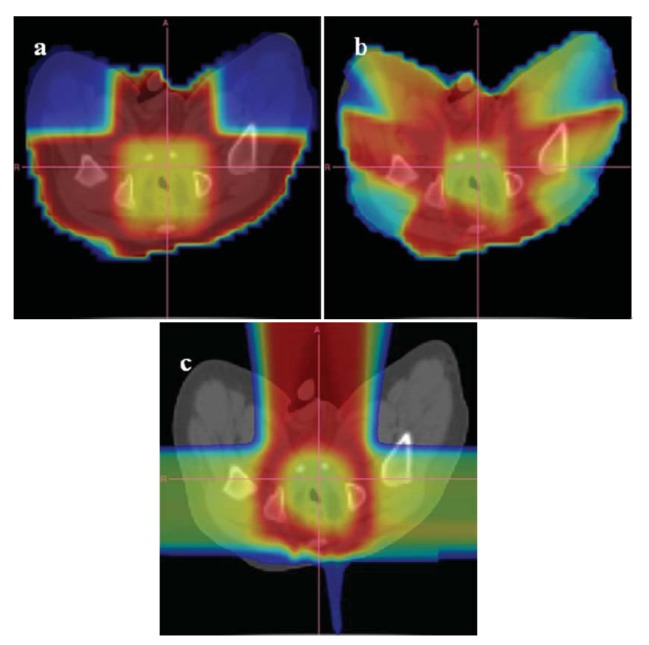
Distribution of risk equivalent dose (RED) in a 1 year old radiotherapy patient with a rabdomyosarcoma in the prostate. The patient was planned with 3D-conformal photons depicted in (**a**), intensity modulated photons (**b**) and pencil beam protons (**c**). RED is shown in colors and covers a range of 0 to 9 Gy from blue to red.

**Figure 9:**
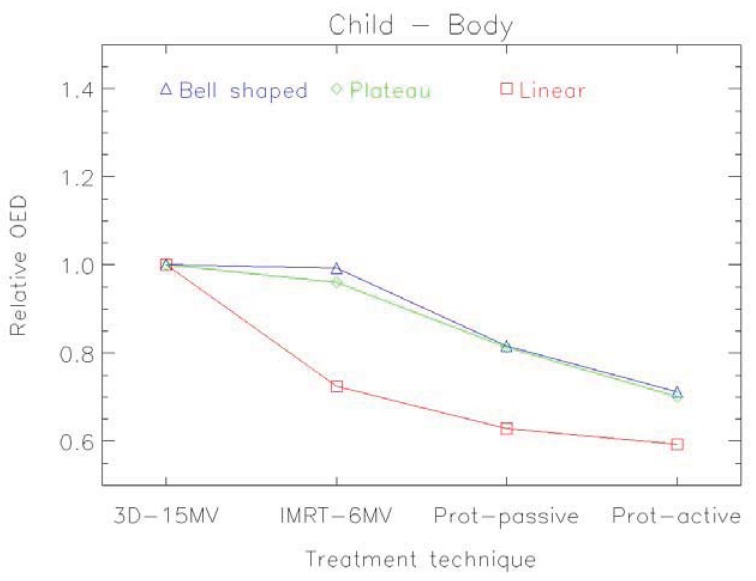
Organ equivalent dose (OED) for the whole body of a one year old radiotherapy patient with a rabdomyosarcoma in the prostate. The OED-ratios relative to 3D-conformal photons are shown for intensity modulated photons, pencil beam protons and scattered protons. Calculations were performed for three different dose-response relationships: a linear (red), a bell shaped (blue) and a plateau (green) one.

An example of the distribution of risk equivalent dose for different treatment plans is shown in Figure 8 and a comparison of OED ratios for different treatment techniques in Figure 9 [61].

## 6. Conclusions

There is increasing concern regarding radiation-related second cancer risks in long-term radiotherapy survivors and a corresponding need to be able to predict cancer risks at high radiation doses. Most information on the dose-response of radiation-induced cancer is derived from data on the A-bomb survivors who were exposed to γ-rays and neutrons. Since, for radiation protection purposes, the dose span of main interest is between zero and one Gy, the analysis of the A-bomb survivors is usually focused on this range. With increasing cure rates, estimates of cancer risk for doses larger than one Gy are becoming more important for radiotherapy patients. Therefore, in this review, emphasis was placed on doses relevant for radiotherapy with respect to radiation induced solid cancer.

Radiation protection models should be used only with extreme care for risk estimates in radiotherapy, since they are developed exclusively for low dose. When applied to scatter radiation, such models can predict only a fraction of observed second malignancies.

Better semi-empirical models include the effect of dose fractionation and represent the dose-response relationships more accurately. The involved uncertainties are still huge for most of the organs and tissues. A major reason for this is that the underlying processes of the induction of carcinoma and sarcoma are not well known. Most uncertainties are related to the time patterns of cancer induction, the population specific dependencies and to the organ specific cancer induction rates. For radiotherapy treatment plan optimization these factors are irrelevant, as a treatment plan comparison is performed for a patient of specific age, sex, *etc*. If a treatment plan is compared relative to another one only the shape of the dose-response curve (the so called risk-equivalent dose) is of importance and errors can be minimized. Therefore, treatment plan comparisons can be performed using mathematical models like organ equivalent dose in combination with epidemiological obtained absolute risk data.
